# Single Implant-Retained Mandibular Overdentures: A Literature Review

**DOI:** 10.7759/cureus.52486

**Published:** 2024-01-18

**Authors:** Dina Elawady, Maya Ali Adam, Hamzah Allam, Ihab Ismail Mahmoud, Ahmed Yaseen Alqutaibi, Ahmed Atef Shon

**Affiliations:** 1 Prosthodontics, October University for Modern Sciences and Arts, Cairo, EGY; 2 Prosthodontics, Taibah University, Madinah, SAU; 3 Removable Prosthodontics, Al-Azhar University, Cairo, EGY; 4 Substitutive Dental Sciences, Taibah University, Madinah, SAU; 5 Prosthodontics, Ibb University, Ibb, YEM; 6 Prosthodontics, Al-Azhar University, Cairo, EGY; 7 Prosthodontics, Al Mouwasat Hospital, Madinah, SAU

**Keywords:** prosthodontics, overdenture, complete denture, single implant, dental implant

## Abstract

The absence of teeth, known as edentulism, poses considerable obstacles in prosthodontic care and greatly affects a person's well-being. Conventional complete dentures frequently lead to problems like instability and insufficient retention, especially in the lower jaw. Fortunately, the introduction of dental implants has transformed the way we approach edentulous patients, as they now offer support and enhanced retention for removable prostheses, thus revolutionizing their treatment. While a consensus exists on using two implants for retaining mandibular overdentures, the associated cost may be prohibitive for economically disadvantaged individuals. As a solution, the concept of single implant-retained mandibular overdentures has emerged, catering to individuals with limited financial resources and complete tooth loss. This review explores the efficacy and suitability of the single implant overdenture approach, along with an overview of treatment options for edentulous patients, including traditional dentures, tooth-supported overdentures, and implant-supported overdentures. The preservation of bone, improvements in functional abilities, and psychological benefits associated with overdentures are discussed. Moreover, various classifications and prosthetic options for implant overdentures, specifically for mandibular cases, are presented. This literature review aims to provide a comprehensive understanding of possible treatment options and focus on the single implant-retained mandibular overdenture approach and its implications in prosthodontic rehabilitation for edentulous patients.

## Introduction and background

Edentulism poses significant challenges in prosthodontic management and can have a profound impact on an individual's quality of life. Although traditional complete dentures have long been used to treat edentulous patients, they frequently present challenges such as instability and poor retention, causing discomfort and anxiety, especially with mandibular dentures [1].

The advent of dental implants has revolutionized the management of edentulous patients by providing support and retention for removable prostheses. However, determining the optimal number of implants for retaining mandibular overdentures is still a topic of ongoing research. There is a consensus that suggests that a standard approach for the edentulous mandible is an implant-retained overdenture with two implants placed in the anterior mandible. Nonetheless, the cost associated with this treatment may be prohibitive for many economically disadvantaged edentulous individuals, particularly in developing countries [2].

In light of this challenge, the concept of a single implant-retained mandibular overdenture has arisen as a viable treatment alternative, specifically for edentulous individuals with restricted financial means or those who are medically compromised and unable to endure invasive surgical procedures to install multiple implants that may necessitate bone grafting [3]. While the single implant overdenture approach may offer economic advantages, it necessitates comprehensive evaluation and investigation to determine its efficacy and suitability. This literature review aims to provide a comprehensive understanding of potential treatment options, with a specific focus on the single implant-retained mandibular overdenture approach and its implications in prosthodontic rehabilitation for edentulous patients.

## Review

Treatment options for edentulous patients

Edentulous individuals have traditionally been treated with complete dentures, which allow them to participate in activities such as speech and eating while also preserving their facial aesthetics. This treatment involves placing a full set of prosthetic teeth that are held in place by the gums and underlying bone to replace all missing teeth. However, complete dentures can be unstable, especially in the mandibular jaw, due to poor retention. This lack of stability can be caused by factors such as changes in the bone structure, reduced muscle tone and saliva production, the absence of natural tooth roots for stability and support, and reliance on physical adhesion with the oral mucosa, which is less strong compared to other retention methods. This has led to criticism of this treatment modality and the recognition of the need for alternative options [4]. Furthermore, rather than preserving the bone, incorrectly planned complete dentures accelerate bone resorption. This may be attributed to the concentration of chewing forces on specific areas of the bone, primarily the outer layer, rather than evenly distributing them throughout the bone. As a result, there is a loss of bone volume and reduced blood flow [5]. These issues, including problems with prosthesis retention, stability, and comfort, can significantly impact functional abilities such as speech, aesthetics, and chewing. Dental implants have revolutionized the treatment options for completely edentulous patients, providing solutions through either fixed [6-8] or removable implant-supported prostheses [9-14]. Even in cases where patients have systemic conditions [15, 16] or require bone grafting prior to implant placement, dental implants have been shown to yield effective outcomes [17-22].

Compared to conventional complete dentures, overdentures have demonstrated improvements in biting force, chewing efficiency, and speech clarity. Moreover, they promote the preservation of the alveolar ridge and offer superior support and retention for the denture [23]. The preservation of the proprioceptive system, which relies on periodontal ligaments, and the psychological acceptance of overdentures further enhance their viability as a valuable treatment option for completely edentulous patients [24]. By distributing masticatory forces more evenly across the roots and denture-supporting tissues, overdentures minimize the impact on these structures, resulting in less alveolar bone loss compared to conventional complete dentures [24].

Overdentures

An overdenture refers to a removable prosthesis that covers natural teeth, tooth roots, and/or dental implants, serving as a complete or partial denture. Various terms, such as overlay dentures, telescoped dentures, tooth-supported dentures, hybrid prostheses, crown and sleeve prostheses, and superimposing dentures, are used to describe this treatment technique [25]. The support provided is a crucial factor, leading to the classification of overdentures into two types: implant-supported overdentures and tooth-supported overdentures. Overdenture abutments can be derived from teeth that are unable to support a fixed or removable partial denture. By reducing these teeth to achieve a favorable crown-root ratio, utilizing the splinting action of the overdenture, and establishing a favorable occlusion spread over a larger region, the strength and support of these teeth can be maintained for a certain period of time [25]. Preserving the remaining teeth, even those with a poor prognosis helps reduce alveolar bone loss and improves denture stability, particularly in the mandible, where mandibular denture stability is commonly problematic [26].

The ability of the mandible to collapse freely into the intercuspal position is attributed to periodontal receptors. In contrast to fully edentulous patients, the proprioceptive nerve terminals in the periodontal ligament transmit sensory information to the neuromuscular system, aiding in the establishment of centric jaw relation for overdentures [23]. The vertical walls of the remaining teeth contribute extra support to the overdenture, and the extent of tooth preparation directly influences the stability it provides. Furthermore, overdenture abutments offer additional denture support. Compared to conventional full dentures, tooth-supported dentures provide support to the overdenture and establish a defined vertical stop for the denture base, resulting in reduced stress on soft tissues and fewer post-insertion complications [26]. The psychological benefits of denture anchoring also contribute to increased patient confidence in social situations, as the presence of retained roots provides a sense of security [26].

Clinical studies conducted on overdenture patients have shown an increased incidence of caries around the abutment teeth. This is attributed to the coverage of teeth by the overdenture, which leads to the absence of a salivary film and the buffering action of saliva around the abutments, thereby increasing the risk of acid attack and susceptibility to caries. Various approaches have been used to manage caries, including protecting the tooth with a metal casting or using topical fluoride. Fluoride gel has been demonstrated as an effective method for preventing cavities on retained overdenture abutments. It should be noted that covering the gum edges of abutment teeth with an overdenture has the potential to cause periodontal disease, leading to gingival hemorrhage and visible inflammation around the abutment teeth. To minimize these issues, meticulous plaque control is recommended [23]. Another drawback is the presence of significant labial undercuts at the abutment teeth, which can interfere with denture insertion. This often requires the removal of excessive acrylic resin from the inner surface of the denture, weakening the denture base and making it more susceptible to fracture [24]. In addition, an overdenture may be bulkier than a full denture, resulting in speech difficulties and compromised esthetics. Furthermore, the significant occlusal pressure exerted on the overdenture can lead to denture base fracture at the abutment area due to wear-induced thinning between the denture base and the supporting natural teeth. A well-designed occlusion can reduce the risk of fracture.

Implant-supported overdentures

Implant-supported overdentures have emerged as a solution to address common concerns such as tooth decay and periodontal disease. Compared to relying on endodontic, periodontal, and prosthodontic procedures to preserve teeth as overdenture abutments, the use of implants has proven to be more cost-efficient and predictable [27]. The placement of dental implants has evolved to become a less time-consuming, aesthetically appealing, and minimally invasive procedure for the restoration of missing dentition [28].

One of the advantages of implant-supported overdentures is their ability to preserve the surrounding bone. In the case of anterior mandibular bone beneath an implant overdenture, it has been observed that bone resorption may reach up to 0.5 mm over a five-year period, with long-term resorption remaining relatively constant at 0.1 mm per year. This level of bone resorption is significantly lower than what is typically observed in edentulous ridges and is comparable to the condition seen with overdentures supported by natural teeth. Consequently, dental implants help protect the bone and prevent further loss [27].

Prosthetic options for implant overdentures

Various classifications have been proposed to categorize implant placement, whether in the form of fixed or removable prostheses. Misch introduced a classification system consisting of five prosthetic solutions for patients with implant-supported prostheses. The first three solutions involve fixed prostheses, which can be either cemented or screw-retained and can replace partial or complete dentitions. The choice of solution depends on the number of structures being replaced, both in terms of hard and soft tissues, as well as the aesthetic requirements of the prosthesis. The remaining two solutions are detachable overdenture prostheses, which differ in terms of implant support, with fully removable overdentures being considered an acceptable treatment option [29].

Furthermore, Misch proposed five well-defined treatment alternatives specifically for implant-supported mandibular overdentures in completely edentulous patients. The first treatment option (OD1) involves placing two implants in the canine regions without connecting them through a superstructure. The most commonly used connection design in this case is an O-ring configuration. The second treatment option (OD2) involves placing implants in the canine regions and splinting them together with a superstructure without a distal cantilever. This results in lower loading forces on the two anterior implants when they are splinted with a bar, as compared to individual implants. In the third treatment option (OD3), three root-form implants are inserted and joined with a superstructure bar, again without a distal cantilever. The additional implant helps reduce the flexure of the superstructure. The fourth treatment option (OD4) involves placing four implants in the canines and first premolar regions, providing sufficient support to include a distal cantilever of up to 10 mm on each side if stress factors are low. The fifth treatment option (OD5) consists of placing five implants, with an additional implant at the midline, and cantilevering the superstructure distally up to a maximum of 2.5 times the anterior-posterior spread [29].

Implant overdentures can be further classified based on the type of support they rely on, namely predominantly mucosa-supported, implant-mucosa-supported, and implant-supported overdentures. The term "mucosa-supported" is used to describe overdentures retained by studs or magnetic attachments, typically involving two implants. On the other hand, "implant-mucosa-supported" refers to overdentures retained by a resilient bar attachment, usually involving four implants [30]. When four or more implants are used to provide complete support, the prosthesis is considered entirely implant-supported, with no contribution from the surrounding mucosa. In certain cases, a distally expanded cantilever bar has been suggested to enhance the retention of the distal component. However, this may lead to increased stress on the implants during mastication [31].

Another study defined implant-retained overdentures in terms of being entirely implant-supported, predominantly implant-supported, implant-retained, or implant-retained and soft tissue-supported. The term "implant-supported" was primarily used to describe overdentures held in place by multiple bar segments or more than two ball attachments without a cantilever extension, as limited movement is expected in both cases due to the absence of a rotational axis [32-34]. In the case of an implant-mucosa-supported prosthesis, load sharing occurs between the implants and the distal extension mucosa. This often involves the use of fewer implants, typically two interforaminal implants, and allows for movement of the prosthesis under functional loads. The attachment's durability, such as with stud attachments, and/or the presence of a spacer layer between the superstructure and the attachment, as in bar or resilient telescopic attachments, enable movement and/or flexion of the distal extension of the denture base, provided that there is intimate contact between the denture base and the load-bearing mucosa. Consequently, the degree of permitted movement and the percentage of load shared between the implant and the mucosa may vary. Implant overdentures offer several advantages over completely implant-supported fixed dentures, such as requiring fewer implants, resulting in lower costs for the patient [35, 36]. Additionally, overdentures are typically easier to repair compared to fixed restorations, provide better esthetics, and offer soft tissue support for the facial appearance of many edentulous patients [37]. Moreover, the ability to remove overdentures at night reduces the impact of para-functional habits and associated strains on the implant support system [38]. Furthermore, the use of robust attachments in overdentures helps minimize stress on the implants and allows for stress release [38]. Implant-supported overdentures also offer the advantage of reducing soft tissue coverage by reducing prosthesis flanges and palatal coverage, leading to increased patient satisfaction and improved taste sensation [39]. According to the McGill consensus statement in 2002, the initial treatment option for a completely edentulous mandible is the placement of two implants to support an overdenture, as this option provides superior stability and speech compared to conventional full dentures [40].

However, there are certain drawbacks associated with mandibular implant-retained overdentures. One significant concern is related to the psychological impact on patients, as the removable nature of the prosthesis may affect their perception and acceptance compared to fixed implant-supported prostheses [29]. Another issue is the requirement of a minimum interarch space of 10 mm to accommodate the denture teeth, acrylic resin foundation, and attachment retainers for implant-retained overdentures [41, 42]. Long-term maintenance is also a notable challenge for mandibular overdenture wearers, as attachments like O-rings and clips may need frequent replacement due to wear and tear [43-45]. Additionally, the posterior ridge in implant-supported overdentures tends to resorb faster than the anterior bone, necessitating regular relining to maintain a proper fit [46]. Food impaction is another concern with mandibular overdentures, as the flanges do not reach the bottom of the mouth in the rest position, potentially leading to discomfort [42].

Mandibular single implant-retained overdentures

According to previous studies, there were no significant changes in patient satisfaction or clinical or radiographic conditions when comparing the use of two or four implants for overdenture treatment [2]. However, the financial cost associated with therapy is an important consideration in treatment selection; as the number of implants increases, so does the cost [3]. Consequently, there is a need for a more cost-effective treatment alternative, such as an overdenture retained by a single implant. Single implant-retained overdentures have gained popularity in recent years due to their lower costs, minimal tissue stress, potential surgical advantages, reduced associated morbidity, and decreased post-surgical maintenance [47-50].

Multiple studies have demonstrated the effectiveness of single implant-retained overdentures, showing no significant differences in survivorship between a single implant and two implants when used with a full mandibular overdenture [49]. For elderly edentulous individuals seeking improved oral health-related quality of life (OHRQoL) and chewing abilities, a single implant placed in the midline of the mandibular arch is recommended as a viable treatment option [51]. Additionally, individuals experiencing pain and functional issues with their traditional mandibular dentures can benefit from this treatment [52]. Furthermore, single implant-retained overdentures have shown comparable patient satisfaction to two implant-retained overdentures while offering the advantages of reduced cost and shorter treatment time [49].

Recent research has indicated that the lateral stresses on the abutments of mandibular overdentures held by one or two implants are similar [53]. Clinicians have suggested that as the number of implants increases, the maximum strain value in the peri-implant bone decreases, and the strain distribution becomes more evenly distributed. This is based on the concept that adding additional anchoring and support implants reduces the force carried by each implant, resulting in less strain on the bone. However, under different loading conditions, the strain value in peri-implant bone has been observed to increase with the number of implants [54]. In contrast, single implants exhibit minimal strain in the peri-implant bone, low stress in the abutments, and no detrimental strain concentration in the bone surrounding the lone implant.

Comparing overdentures retained by single and two implants, it has been found that under vertical load, single implant overdentures rotate over the implant from side to side without a significant increase in peri-implant bone strain. In contrast, two implant-retained overdentures show more apparent rotation around the fulcrum line passing through the two implants, and the maximum equivalent stress in the abutments is higher [54, 55].

The use of a single implant with a ball attachment has been clinically proven to be a satisfactory treatment for retaining mandibular overdentures, as it increases denture stability, oral health-related quality of life, and masticatory function [56, 57]. Large ball attachment systems, such as titanium nitride-coated patrices and plastic matrices, have been found to provide higher retentive forces for mandibular single-implant overdentures, as well as favorable wear behavior and clinical performance [58, 59]. When comparing the Locator attachment to ball attachment systems for restoring single implant-retained mandibular overdentures, the Locator attachment has been found to be superior, as other attachment systems are prone to wear and loss of retention, leading to significant maintenance costs [59, 60].

Mandibular single-implant overdentures, when used with an early loading strategy and implants of various diameters and attachment methods, have been shown to be a viable treatment option for older edentulous individuals [58]. However, there are risks associated with inserting a single implant in the midline of a damaged atrophic mandible, such as mandibular fracture and proximity to the lingual artery [61]. One of the common complications with single implant-retained overdentures is denture fracture and the need for more prosthetic maintenance. The concentration of stresses within the denture bases over the implants may explain why there is a relatively higher incidence of denture fractures in single-implant overdentures. Greater fracture risk may also be associated with the tendency of the denture to fulcrum over a single implant when the mucosa atop the remaining ridge compresses with denture wear or the ridge resorbs, necessitating ongoing monitoring over time. However, several trials have shown no substantial difference in the frequency of denture base fractures between overdentures retained by a single implant and those retained by two implants [62]. To reduce the risk of denture breakage, a metal-strengthened single implant mandibular overdenture with a locator attachment as a retention device has been suggested. The metal-reinforced structure within the acrylic resin foundation provides increased stiffness, preventing denture fracture [63].

There is a possibility that the forces exerted on an overdenture supported by a single implant are greater than those exerted on a multiple implant-retained/supported overdenture, which may increase the risk of loss of osseointegration [1]. Biomechanical studies have shown that single-implant overdentures exhibit similar biomechanical characteristics to two-implant overdentures in terms of lateral forces on the abutment and denture base movements under functional molar loads [53]. The success of single implant-retained overdentures is also influenced by patient selection. Since a single implant cannot provide significant support and retention for the entire arch, the stability of the overdenture relies heavily on the support provided by the alveolar ridge tissue [64].

When planning the treatment for a single implant-retained full denture, it is crucial to emphasize that the prosthesis should be tissue-borne. The stability and retention of the denture are achieved by contacting key stress-bearing sites, such as the residual ridge and the buccal shelf. Engaging the retromylohyoid area can improve the lateral stability of the mandibular prosthesis, and the use of an attachment in a single implant-supported overdenture enhances retention [64]. In cases with significantly atrophied ridges, the absence of resistance to lateral and rotational stresses can compromise stability and chewing efficiency. Therefore, stud attachments such as the Locator may provide better stability and retentive force compared to magnetic attachments [59].

In individuals with inadequate alveolar tissue support, the quality of overdenture occlusion, particularly balanced occlusion, is crucial. To avoid harmful lateral and rotational stresses during function, occlusal contacts should be balanced in all directions [65, 66]. A reduced dental arch overdenture is proposed based on the theory that reducing the number of posterior teeth in an implant-supported overdenture can prevent crestal bone loss around the implants during the healing phase [65, 66].

The utilization of single symphyseal implants to rehabilitate edentulous mandibles was first reported by Naert et al. in 1991 [67]. In the same year, Cordioli et al. conducted a five-year prospective analysis of 21 senior patients treated with single implant-retained overdentures, reporting a 100% success rate with low yearly radiographic bone loss [68]. This treatment technique offers a successful, cost-effective, and practical option for patients with mandibular ridge resorption, for whom wearing traditional full dentures is challenging. Several investigations have demonstrated the success and clinical viability of single implant-retained overdentures [68]. However, these studies have shown variations in procedures and have been associated with various difficulties. Systematic reviews conducted by Srinivasan et al. and Batista et al. assessed the clinical viability of single implants with overdentures and found that their survival rates were comparable to those of two implants with overdentures [69,70]. Nogueira et al. conducted a comprehensive evaluation and reported that single implant-retained overdentures improved patient satisfaction compared to standard full denture treatment [71]. Several studies demonstrated that single implant-retained overdentures outperformed traditional two implant-retained overdentures in terms of implant success and marginal bone loss [72-79].

The studies reported the use of implants with an average length of 10 mm, ranging from 7 mm to 15 mm. The sizes of the implants used varied from 3.75 mm to 8 mm throughout the trials. Alsaheeba et al. observed a 100% success rate in implants with diameters of 4 mm and 8 mm, compared to implants with a diameter of 2.75 mm, which had a 75% survival rate [75]. Various surface treatments were employed, including air abrasion, acid etching, sandblasting, and acid etching, with studies reporting a 100% implant survival rate [78, 79]. According to Liddelow et al., oxidized implants had a 100% survival rate, while all machined implants used in the study failed [74, 75]. The total failure rate per 100 implant years was 6.03, and the estimated five- and 10-year survival rates were 91.93% and 83.95%, respectively [76].

For immediate loading, implants with an insertion torque of 45 Ncm were considered suitable in some reports, and implants with an implant stability quotient (ISQ) of 60 were deemed appropriate for immediate loading [74-78]. Immediate loading was used in five experiments, while early loading was performed in three trials at six weeks postoperatively. The remaining trials employed a standard (delayed) loading strategy with durations ranging from two to four months [68, 73, 79, 80-87]. The survival rates varied depending on the loading protocol.

Nogueira et al. reported a survival rate of 100% for immediate loading and early loading protocols, while delayed loading had a survival rate of 96.6% [71]. Alsaheb et al. reported a 100% survival rate for immediate loading and early loading and a 92.8% survival rate for delayed loading [75]. The success rates reported in the studies varied between 88% and 100%, with the majority of studies reporting success rates above 90% [68-87].

In summary, single implant-retained overdentures can be a successful treatment option for patients with mandibular ridge resorption who have difficulty wearing traditional full dentures. The success rates of single implant-retained overdentures are comparable to those of two implant-retained overdentures. Factors such as patient selection, implant length and diameter, surface treatment, loading protocol, and occlusal considerations play important roles in the success and stability of single implant-retained overdentures. It is important to consult with a qualified dental professional or implantologist to determine the best treatment plan for your specific situation. They can evaluate your oral health, bone structure, and other factors to provide personalized recommendations and discuss the potential risks and benefits of single implant-retained overdentures. Table 1 summarizes the differences between a conventional complete denture, a single implant overdenture, and a multiple implant overdenture.

**Table 1:** The differences between a conventional complete denture, a single implant overdenture, and a multiple implant overdenture

Factors	Conventional complete denture	Single implant-retained overdenture	Multiple implant-supported overdenture
Retention	Dependent on physical means of retention and can use adhesives	Improved retention with the use of an attachment screwed to the implant	Enhanced retention due to multiple attachments used with implants
Number of implants required	None	One implant (placed at midline)	Two or more implants
Patient satisfaction	Varies depending on individual experience	Generally higher satisfaction due to improved stability	High satisfaction due to improved stability and functionality
Biting force	Reduced biting force compared to natural dentition	Improved biting force compared to conventional dentures	Enhanced biting force, closer to the natural dentition in cases with four or more implants
Maintenance	Regular cleaning of dentures and oral hygiene	Regular cleaning of dentures and oral hygiene, periodic implant and attachment maintenance	Regular cleaning of dentures and oral hygiene, periodic implant and attachment maintenance
Time needed for construction	It typically requires fewer visits and shorter construction time than implant-supported options.	Requires multiple visits and longer construction time compared to conventional dentures	Requires multiple visits and longer construction time compared to conventional dentures
Cost	Lower cost compared to implant-supported options	Moderate cost, considering the cost of the implant and denture	Higher cost due to multiple implants and dentures

Attachments used in overdentures

A retainer comprises a metal receptacle and a closely fitting part, with the former (the female matrix component) typically housed within the natural or expanded contours of the abutment tooth crown and the latter (the male patrix component) attached to a pontic or denture framework [14]. Attachments offer several advantages, including enhancing the retention of tooth and implant-supported overdentures and providing both vertical and horizontal stability, thereby increasing their longevity [88]. The intermittent contact of a resilient attachment may also stimulate the underlying tissues and help resolve abutment alignment issues [88].

However, it has been shown that the incorporation of certain types of attachments in the prosthesis can impose strains on the supporting components [89]. Several factors need to be considered when deciding whether to splint or unsplint implants using attachments. These factors include the anatomical situation of the mandible, desired level of retention, ability to maintain hygiene, interarch distance, parallelism of the implants, and cost considerations [90-92].

Treatment planning and selection of overdenture attachments

Amount of Retention Needed

The retentive properties of attachments are influenced by various factors, such as maximal retentive force, range of retention, retention energy, and exhaustion behavior [93]. It is, therefore, crucial to comprehend the retentive and stabilizing qualities of attachments under different dislodging patterns. The release time is a significant factor when evaluating attachment retentive characteristics; attachments with slower release times exhibit lower retentiveness. For instance, bar attachments have a quicker release time compared to magnet attachments, rendering them more retentive [93]. The design of overdenture attachments and the forces involved in dislodgment can significantly affect the stress and strain around implants [94]. Stronger attachment retention leads to higher transmitted stresses to implants. Hence, understanding the retentive and stabilizing qualities of attachments is crucial in selecting the appropriate overdenture attachment type. In a study comparing magnetic and stud overdenture attachments, stud attachments demonstrated superior retentive and stabilizing forces compared to magnetic attachments [95].

The retentive force of Locator, ball, and magnetic attachments in mandibular implant-retained overdentures is provided by mechanical interlocking, frictional contact, or magnetic forces of attraction between the patrix and matrix components. In terms of retention, magnet attachments have shown the highest value, followed by ball and socket attachments [96-99]. It is widely accepted that bar and clip combinations offer greater retention values compared to ball or magnetic attachments [95]. Telescopic retainers have also been proven to be beneficial in implant-retained prostheses due to the frictional fit between the crown and sleeve, which ensures good retention [100]. However, it has been observed that telescopic crowns may lose retention over time due to changes in surface characteristics and metal abrasion [101].

Recently, a combination of a main screw-held framework connected to the implants and a secondary detachable superstructure with parallel friction pins and swivel latch attachments has been used to enhance retention [88]. When analyzing retention and stability in single implant overdenture scenarios, only Locator and ball attachments were found to provide adequate vertical retention. Furthermore, when comparing three widely spaced implants to three narrowly spaced implants, O-ring attachments showed reduced resistance to dislodgment, while extracoronal resilient attachment (ERA), Locator, and ball attachments exhibited greater resistance to dislodgment, indicating that widely spaced implants result in greater resistance to dislodgment for these attachments. However, when considering posterior dislodging pressures, only ball attachments offered sufficient resistance [56]. The horizontal stability of an overdenture prosthesis can be affected by the robustness of the attachment system. In comparison, Locator and ball attachments demonstrated greater stability than ERA and O-ring attachments, allowing for more flexibility in the matrix-patrix interaction and rotational movements [102].

The role of Locators in ensuring proper retention and correct seating of implant-supported overdentures is crucial. The connection of the Locator includes a skirt that wraps around the denture components, facilitating the precise alignment of the permanent mating component on the implant. The self-aligning functionality of the Locator acts as a guide plane for single implant-detachable overdentures [103].

Maxillomandibular Relationship and Interarch Space

Sufficient vertical and buccolingual space is required for the placement of attachments [104]. It is essential to encase attachments in an appropriate layer of acrylic to prevent the weakening of the denture [105]. Resilient attachments often require a significant interarch space and may contribute to posterior mandibular resorption due to vertical denture movement. On the other hand, non-resilient stud attachments allow for mobility, while rigid attachments are utilized when interocclusal space is limited [106].

In terms of interarch distance requirements, reports indicate that bar attachments necessitate a minimum distance of 13 mm, whereas Locator attachments require 8.5 mm. This discrepancy is primarily attributed to the fact that the bar attachment obtains retention either directly from the bar through a clip or stud attachments attached to the body of the bar. In contrast, the male half of the Locator attachment achieves retention by engaging the female component attached to the implant, and this internal retention feature has a modest profile. The design of the Locator attachment benefits from a low height requirement (3.7 mm) and a larger cross-section to ensure strength. Additionally, the shorter height of this attachment is advantageous in situations where interocclusal height is limited [107, 108].

Inter-implant Distance and Parallelism

The parallelism of implants is considered a critical factor in selecting the most suitable overdenture attachment type [92]. Optimal retention can be achieved when all implants are placed as parallel to each other as possible [109]. Excessive divergence between implants can lead to increased rates of wear. There are five types of retainers with stud attachments positioned at different angles to the retaining dowels, ranging from 0 to 20 degrees. The male component of the attachment features a hemispherical head that allows for ball and socket rotation and includes a pressure release groove to facilitate denture fitting [110]. The Locator attachment can also accommodate divergent implants up to 20 degrees. Various options for abutment heights, angulation correction, and retention levels are available to assist clinicians and laboratory technicians in creating the most suitable overdenture restoration for each patient [111].

Cost-Effectiveness

Precision attachments are considered to be more precise than semi-precision attachments and offer advantages such as reduced wear on the abutment and the availability of standard parts that can be easily inserted and maintained. In contrast, semi-precision attachments are more cost-effective [112, 113].

Compared to bar and clip-type implant overdentures, the use of stud attachments is a simpler and more cost-effective procedure. O-ring or ball attachments are particularly popular and widely available for implant overdentures using various implant systems due to their affordability. Solitary ball attachments are reported to be less expensive, less technique-dependent, and easier to clean than bar attachments [114].

One specific type of attachment suitable for Locator abutment platforms is the Go-Direct Prosthetic System (GPS) attachment. The GPS attachment is a low-profile cap attachment that provides rotational and vertical stress-breaking capabilities. It differs from the Locator attachment in that it does not require a specific tool for component insertion and is also less expensive [115].

Maintenance and Oral Hygiene

Stud attachments offer a hygienic advantage by minimizing conflicts between vestibular flanges apical to the abutment, exposing the bulge, and creating a more sanitary environment [105]. Additionally, telescopic overdentures are relatively easy to maintain, similar to ball attachments, as they lack difficult-to-access areas for maintenance. Moreover, telescopic overdentures have the added benefit of experiencing fewer technical issues compared to ball attachments [116].

Patients with bar attachments who have poor oral hygiene are more prone to developing mucosal hyperplasia beneath the bar and experiencing inflammation of the soft tissue surrounding the implants. Therefore, patients with inadequate oral hygiene practices are not suitable candidates for bar attachments unless they are committed to a suitable oral hygiene routine. However, in cases where a rigid prosthesis is necessary but hygiene constraints prevent the use of a fixed, detachable prosthesis, the use of a milled bar with spark erosion technology is recommended. This design offers improved hygiene procedures facilitated by the removable superstructure and enhanced facial and dental esthetics achieved through the use of a dental flange [117].

Patient Satisfaction

Patient satisfaction with overdentures is influenced by various factors, including patient preferences, chewing comfort, phonetics, esthetics, implant retention, the fit of prosthetic components and attachments, and denture precision [118]. A prospective crossover clinical study investigated patient satisfaction and its correlation with attachment retention, revealing that patients preferred attachments with higher retention, and an effective retentive force typically ranged from 8 N to 10 N [119].

Stud attachments offer the advantages of improved esthetics and patient comfort, especially for individuals who cannot tolerate palatal coverage, which can trigger the gag reflex. Ball attachments often receive higher patient satisfaction ratings and are associated with fewer soft-tissue or mechanical issues compared to bar and magnet attachments [120, 121]. Magnet attachments have been found to exhibit inferior chewing efficiency compared to ball attachments due to their weaker stability during functional activities [122, 123].

Prosthetic maintenance and complications have a significant impact on patient satisfaction. In comparisons of different overdenture attachments, magnet attachments were found to have the highest frequency of prosthetic maintenance and issues related to wear and corrosion, leading to reduced patient contentment [124]. Other common maintenance needs include clip loosening in bar attachments and matrix loosening in ball attachments. There have been debates regarding whether bar or ball attachments require more maintenance, with some studies reporting higher prosthetic care and problems with ball attachments [125], while others found that bar attachments required more maintenance [126]. Additionally, replacing clips or repairing overdentures in bar attachments was often more time-consuming than in ball attachments. The use of newly designed elliptical gold matrices in ball attachments, featuring broad wings to prevent separation from the denture base, has contributed to a reduction in complications and maintenance requirements.

Both Locator and magnet attachments have demonstrated the ability to significantly enhance patient satisfaction and masticatory efficiency in single implant-retained overdentures [127]. The Locator attachment outperformed the magnet attachment in terms of perceived chewing ability, although no significant difference was observed in objectively evaluated masticatory efficiency between the two types of attachments. Moreover, the installation of both attachment types resulted in substantial improvements in overall happiness, comfort, speech, chewing ability, and retention for patients. However, it should be noted that Locator attachment systems have similar prosthodontic maintenance requirements as other attachment systems, and while most prosthesis-related issues are easily repairable, patient dissatisfaction may arise due to the associated costs of upkeep [115].

Status of the Antagonistic Jaw

According to a consensus statement from McGill University, the choice of implant placement for implant-supported overdentures requires consideration of the opposing arch [104]. In cases where a maxillary full denture opposes the overdenture, stud attachments, rather than bar attachments, are preferred to avoid excessive forces that could destabilize the maxillary denture [128].

The Locator Attachment System, introduced in 2000 by Zest Anchors, consists of a self-aligning double-retention cylinder with inner and outer retention surfaces. The attachment includes a male component, which is an implant screw-metallic abutment, and a female component, which is a metallic cap coated with nylon of various colors indicating different retention capacities and attached to the denture [129]. The nylon attachments come in different varieties, with internal and external retention for well-positioned implants (transparent, pink, and blue) and exterior retention for non-parallel implants (green, orange, and red) [129]. The Locator attachments can accommodate divergent implants and rectify parallelism [129].

Locator attachments are suitable for complete or partial dentures supported by endosseous implants or natural tooth roots in the mandible or maxilla [130]. The choice of Locator attachments depends on their location, with mandibular placement being simpler than maxillary placement [131]. These attachments require minimal interarch space, with a recommended minimum of 8.5 mm of vertical space and 9 mm of horizontal space for implant-supported overdentures [132]. Locator attachments are particularly useful when there is insufficient space for ball attachments and can address various prosthesis-related problems, such as over-contoured prostheses, excessive occlusal vertical dimension, fractured teeth adjacent to the attachments, attachment separation from the denture, prosthesis fracture, and patient dissatisfaction [132].

One limitation of Locator attachments is their inability to provide completely rigid connections, as they allow for rotational dislodgement [131]. However, the swiveling capacity of the denture cap over the male component accommodates natural occlusion movements and the pliancy of the supporting soft tissue, ensuring a secure fit for the overdenture during chewing [129]. The rounded and cylindrical nature of the Locator attachment provides a strong connection, transferring forces and movements directly to the implant [131]. Locator attachments are not suitable for free-end saddle situations and can also be used in tooth-supported prostheses [133].

Implants with a minimum diameter of 3.3 mm are recommended for Locator attachments, and implant centers should be spaced at least 6.5 mm apart to accommodate the 5.5 mm broad metal housings [134]. Proper positioning of the Locator connection in a gingival thickness of less than 4 mm is essential to minimize excessive stresses on the implant [134]. The primary advantage of Locator attachments is their ability to adjust retentive pressures using color-coded nylon housings, and their self-aligning feature facilitates easy and quick restoration implantation [135-137].

While both Locator and ball attachments have shown comparable results in some studies, the use of Locator attachments has been associated with soft tissue discomfort and peri-mucositis in some patients, which can be resolved through rebasing or relining of the prostheses [136]. In terms of prosthodontic problems and maintenance of oral function, the Locator system has been found to produce better clinical outcomes compared to ball and bar attachments in totally edentulous individuals [138]. However, Locator attachment systems require more maintenance due to frequent matrix activation and aftercare [138].

The use of Locator attachments for the immediate loading of two implants to support a mandibular overdenture has been found to be a suitable therapeutic strategy, with marginal bone level changes similar to those observed with delayed loading approaches. In vitro investigations have shown that the retentive properties of Locator attachments diminish after repeated pulls, but they still retain significant retention for over 110,000 cycles [137].

One notable drawback of Locator attachments is the time-consuming laboratory procedure and higher cost compared to other attachment systems. Additionally, Locator attachments do not provide splinting [139-141].

## Conclusions

The concept of a single implant-retained mandibular overdenture has emerged as a promising and cost-effective treatment option for completely edentulous individuals with limited financial resources and those who are medically compromised. It addresses the issues of instability and inadequate retention commonly experienced with traditional complete dentures, particularly in the lower jaw. By utilizing a single implant to support a removable prosthesis, this approach significantly improves stability, retention, and chewing efficiency, offering functional and esthetic benefits. However, careful evaluation and investigation are necessary to determine the suitability of this approach for each case, considering factors such as bone quality, occlusion, and patient expectations.

Despite the economic advantages of the single implant overdenture approach, further research is required to assess its long-term success rates, patient satisfaction, and impact on oral health-related quality of life.
